# Supplementary material for: Conservation paleobiology on Minami-Daito Island, Okinawa, Japan: anthropogenic extinction of cave-dwelling bats on a tropical oceanic island
Source: PeerJ. 2022 Jan 27;10:e12702. doi: 10.7717/peerj.12702 (PMC8801181; doi:10.7717/peerj.12702)
Supplement: Supplemental Information 2 [file peerj-10-12702-s002.docx]

**Table S2**. Summary of radiocarbon ages (calBP) of a fossil guano reference and Minami-Daito samples.

| Sample ID | Material Type | Lab Code | uncalibrated BP | | calBP (95.4% confidence interval) | | | δC (‰, VPDB) |
| --- | --- | --- | --- | --- | --- | --- | --- | --- |
|  |  |  | Mean | SD | Median | Lower | Upper |  |
| F170401g1 | Fossil guano (R4) | S-10568 | 1162 | ± 19 | 1070 | 1161 | 1179 | -25.6 |
|  |  |  |  |  |  | 1050 | 1128 |  |
|  |  |  |  |  |  | 978 | 1034 |  |
| Y180119g2 | Fecal pellet-like sample | S-10569 | 4112 | ± 38 | 4640 | 4748 | 4821 | -22.6 |
|  |  |  |  |  |  | 4521 | 4730 |  |
|  |  |  |  |  |  | 4453 | 4462 |  |
| H180120g5-u | Guano-like deposit | S-10567 | 4217 | ± 22 | 4746 | 4806 | 4847 | -27.8 |
|  |  |  |  |  |  | 4700 | 4759 |  |
|  |  |  |  |  |  | 4650 | 4673 |  |
| H180120g5-m | Guano-like deposit | S-10566 | 3991 | ± 21 | 4478 | 4464 | 4521 | -28.0 |
|  |  |  |  |  |  | 4415 | 4451 |  |
| H180120g5-b | Guano-like deposit | S-10565 | 3979 | ± 22 | 4472 | 4465 | 4520 | -27.1 |
|  |  |  |  |  |  | 4410 | 4451 |  |
